# Modified frontolateral partial laryngectomy operation: combined muscle-pedicle hyoid bone and thyrohyoid membrane flap in laryngeal reconstruction

**DOI:** 10.7497/j.issn.2095-3941.2013.02.007

**Published:** 2013-06

**Authors:** Dian Ouyang, Tian-Run Liu, Yan-Feng Chen, Jian Wang

**Affiliations:** 1 Key Laboratory of Oncology in Southern China, Guangzhou 510060, China;; 2 Department of Head and Neck, Sun Yat-sen University Cancer Center, Guangzhou 510060, China;; 3 Department of Otorinolaryngology Head and Neck Surgery, The Sixth Affiliated Hospital of Sun Yat-sen University, Guangzhou 510655, China;; 4 Department of Anesthesia, Sun Yat-Sen University Cancer Center, Guangzhou 510060, China

**Keywords:** Hyoid bone, reconstruction, laryngeal cancer, flap, operation

## Abstract

**Objective:**

Laryngeal reconstruction is needed to preserve laryngeal function in patients who have undergone extensive vertical or frontal partial laryngectomy. However, the procedure remains a difficult challenge. Several reconstruction techniques have been described, but these techniques pose risks of complications such as laryngeal stenosis. This study aimed to evaluate the postoperative course and functional outcomes of a new technique that combined a muscle-pedicle hyoid bone and a thyrohyoid flap during laryngeal reconstruction after tumor resection.

**Methods:**

Four patients underwent extensive vertical partial or frontal partial laryngectomy for cancer. After tumor resection, laryngeal reconstruction was performed using the proposed technique. Postoperative recovery time, complications, and oncologic results were evaluated.

**Results:**

The four patients were successfully treated with the proposed technique. No dyspnea, dysphagia, or death occurred during the postoperative course. Decannulation was performed after a median of 3 days. The average postoperative hospital stay was 7 days. Short-term postoperative functional recovery was normal. No laryngeal stenosis or tumor recurrence was observed in any of the four patients after a follow-up period of more than 24 months.

**Conclusion:**

The combination of the muscle-pedicle hyoid bone and the thyrohyoid flap is a reliable procedure for laryngeal reconstruction after extensive vertical partial or frontal partial laryngectomy.

## Introduction

Laryngeal reconstruction after extensive vertical partial or frontal partial laryngectomy is often challenging. Several reconstruction techniques have been reported. Based on these methods, we considered that a technique using a combined muscle-pedicle hyoid bone and thyrohyoid membrane flap might provide a bony structural support for the remaining thyroid cartilage framework. This technique could also be used for mucosal repair with the fascial thyrohyoid membrane.

Laryngeal reconstruction may be classified in different groups, including laryngeal lumen resurfacing and maintenance as well as glottic and vestibular reconstruction. Different flaps and grafts have been used to construct intraluminal prostheses and skin flaps, mucosal flaps, skin and mucosal grafts, perichondrium and muscle flaps, and cartilage flaps^1^^-^^8^. A muscle-pedicle hyoid bone flap is commonly used in laryngeal reconstruction after vertical partial or frontal partial laryngectomy. The first use of a hyoid bone to repair laryngotracheal stenosis was reported by Looper in 1938^9^. In 1960, Bennett *et al.*^10^ used an autologous hyoid bone graft to treat subglottic stenosis. Alonso *et al.*^11^ also used a hyoid bone in their experimental study of dogs in 1975. Finnegan^12^ was the first to report the use of a hyoid bone combined with a muscle-pedicle flap in dogs. Ward *et al.*^13^ used a muscle-pedicle hyoid bone flap to treat laryngotracheal stenosis. In 2004, Cansiz^14^ used a muscle-pedicle hyoid bone flap to repair laryngotracheal stenosis in 23 patients. However, these studies have revealed a risk of laryngeal stenosis (or failure of reconstruction) ranging from 4% to 32%.

We considered that a muscle-pedicle hyoid bone could provide an effective bony structural support. However, we need to determine whether or not the use of a hyoid bone flap can improve the success rate of reconstruction after vertical partial or frontal partial laryngectomy. The ideal laryngeal reconstruction provides a firm framework to maintain the airway and facilitate re-epithelialization in the laryngeal cavity^1^. Hence, we combined a muscle-pedicle chimeric thyrohyoid membrane with a hyoid bone. The use of the thyrohyoid membrane as local fascia may prevent excessive granulation. In this study, the surgical technique was demonstrated and the results were discussed.

## Patients and methods

### Patients

The research ethics board of the Sun Yat-Sen University Cancer Center approved the study. All of the participants provided a written informed consent. Four male patients (median age 62 years, ranging from 51 to 76 years) underwent the proposed procedure between August 2008 and November 2009. Their clinical features are summarized in **Table 1**. Case one had an anterior commissure and glottic tumors that extended to the subglottis and impaired vocal mobility. This case was defined preoperatively as stage T_2_ but was upstaged to T_4a_ because the cricothyroid membrane was invaded. In case two, glottic tumors were restaged as rT_1b_ after the patient failed to respond to radiotherapy and then upstaged to rT_3_ because of a minor erosion of the thyroid cartilage. Case three classified as stage T_3_ had a glottic tumor extending to the paraglottic space with impaired vocal mobility; anterior commissure was also observed. Case four classified as stage T_4a_ had a subglottic tumor invading cricoid and cricothyroid membranes with normal vocal mobility and anterior commissure. Pathological examination confirmed the diagnosis of a well-differentiated squamous cell laryngeal carcinoma in these four cases. Preoperative examination, including physical examination, laryngoscopy, high-resolution computed tomography or magnetic resonance imaging, and pulmonary function tests were performed in accordance with the NCCN Clinical Practice Guidelines in Head and Neck Cancer 2008.

**Table 1:** Clinical data of patients

Demographics							Treatment				Functional and oncologic outcome						
Patient	Gender	Age (yrs)	Site	CS	PS		Type	ND	Radio		DS	DC	SB	DW	RNT	Recurrence	Follow up (month)
1	M	51	Glottic	T_2_	T_4a_		B	No	Yes		2008.8	5	3	7	7	No	43
2	M	65	Glottic	rT_1b_	rT_3_		A	No	No		2008.9	3	3	6	7	No	41
3	M	76	Glottic	T_3_	T_3_		A	No	No		2009.1	3	3	7	7	Yes	38
4	M	56	Subglottic	T_4a_	T_4a_		B	No	Yes		2009.11	3	3	7	7	No	28

CS, clinical stage; PS, pathology stage; ND, neck dissection; DS, date of surgery; DC, decannulation (day); SB, swallow batter (day); DW, drink water (day); RNT, removal of nasogastric tube (day).

### Operative technique

#### Laryngectomy phase

**Figure 1:**
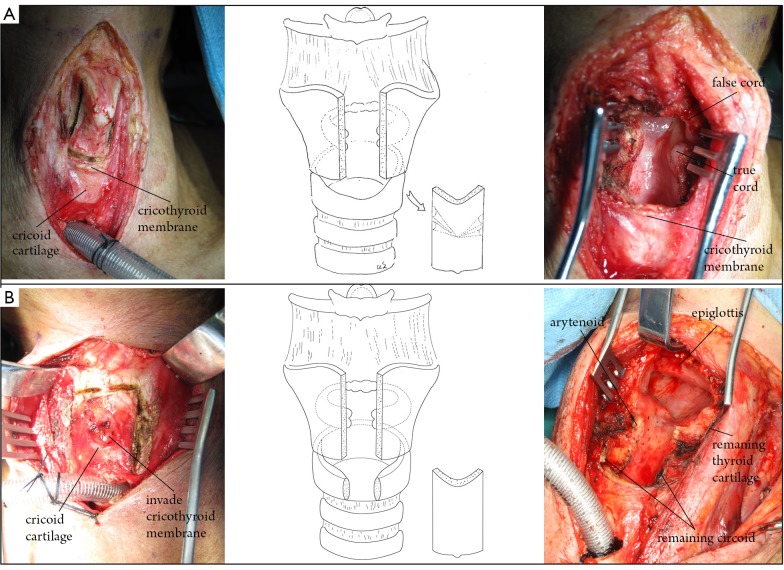
Surgical defect of the larynx after extensive partial laryngectomy. Type A, extended frontal/vertical partial laryngectomy without partial resection of the cricoid cartilage. Type B, extended frontal/vertical partial laryngectomy with partial resection of the cricoid cartilage (frontal partial resection of the cricoid cartilage based on Type A).

Two types of laryngeal framework defects were commonly observed after tumor resection: type A, which was observed after extensive frontal/vertical partial laryngectomy without partial resection of the cricoid cartilage; and type B, which was observed after extensive frontal/vertical partial laryngectomy, including partial resection of the cricoid cartilage (**Figure 1**).

During the procedure, strap muscles were exposed and separated carefully to expose the anterior laryngotracheal wall. Considering the location and the extent of the tumor, the thyroid cartilage was split vertically on the healthy side by using microsaws approximately 5 mm from the edge of the tumor. The frontal partial trachea was cut transversely along the upper edge of the first ring of the trachea (0.5 cm under the edge of the tumor). The incision was curved upward and proceeded vertically to connect to the incision in the thyroid cartilage. The thyroepiglottic ligament was then cut transversely along the upper edge of the thyroid cartilage. The frontal partial laryngeal soft tissue covering the tumor was dissected with the frontal thyroid cartilage on both sides. In the laryngeal cavity, the incision was made at a safe margin of 0.5 cm from the edge of the tumor. If necessary, all of the fold card and the arytenoid cartilage were removed with the tumor. During resection, the thyrohyoid membrane was reserved. Delphian lymph node was removed from the patients and subjected to pathological analysis. Selective lateral neck dissection (levels II, III, and IV) was performed in cases with T_3_ or T_4a_ laryngeal cancer revealing a clinically negative neck.

#### Harvesting flap

The combined muscle-pedicle hyoid bone and thyrohyoid flap should include the hyoid bone with its blood supply, the hyothyroid membrane, and the infrahyoid muscles. The pedicle is an inferior part connected to the upper edge of the thyroid cartilage and the pre-epiglottic space. The suprahyoid muscles were cut along the upper edge of the hyoid bone. The lesser cornu and the greater cornu were cut from the body of the hyoid bone. The infrahyoid muscles and the hyothyroid membrane were kept attached to the hyoid bone and to the upper edge of the thyroid cartilage. The thyrohyoid membrane is a broad, fibroelastic layer attached to the upper border of the thyroid cartilage and the upper margin of the posterior surface of the body and the greater cornua of the hyoid bone. This membrane facilitates the upward movement of the larynx during deglutition. To maintain blood supply, the aforementioned structures were separated from the level of the hyoid body down to the upper edge of the thyroid cartilage. The periosteum and the blood supply of these structures were retained. The flap attached to the upper edge of the thyroid cartilage, which is the origin of the flap pedicle, was also retained.

#### Bone fixation

**Figure 2:**
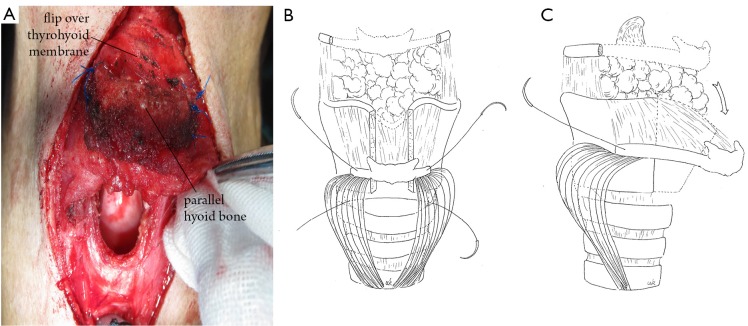
Combined hyoid bone flap in laryngeal framework reconstruction: type A. The hyoid bone was fixed in the glottic position above the remaining thyroid cartilage. A. Surgical view; B. Mortise view; C. Lateral view.

**Figure 3:**
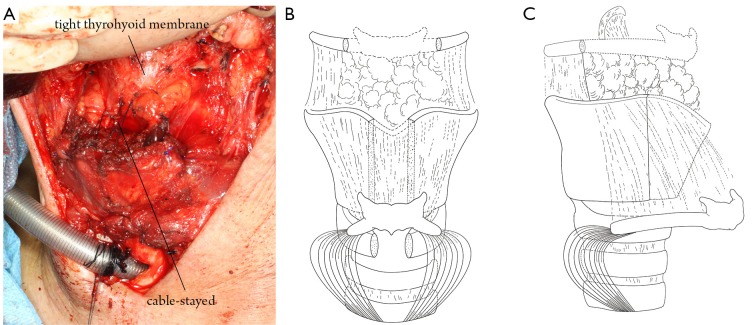
Combined hyoid bone flap in laryngeal framework reconstruction: type B. The hyoid bone was fixed to the remaining cricoid cartilage. A. Surgical view; B. Mortise view; C. Lateral view.

The shoulder pads were removed and the neck was maintained at a supine position. The body of the hyoid bone, pedicled with the attached hyothyroid membrane and infrahyoid muscles, was turned down and attached to the remaining cartilage framework to obtain a stable bony support. In type A defects, the hyoid bone was attached on the remaining thyroid cartilage at the vocal cord level (**Figure 2**). In type B defects, the hyoid bone was attached to the remaining cricoid cartilage (**Figure 3**). Microsaws were used to create peripheral openings, attaching the bony graft to the thyroid or the cricoid cartilage with non-absorbable 2-0 Prolene sutures (Ethicon, Johnson & Johnson Medical, Livingston, UK). The hyoid bone was fixed in a bridge-like fashion across the cricothyroid cartilage edges (**Figure 2**). The laryngeal cavity was slightly expanded when fixing to ensure that the new cartilage framework was stable and provide ventilation for the larynx.

#### Repair of laryngeal mucosal defect

The thyrohyoid membrane, a tough fibroelastic ligament attached to the superior margin of the thyroid cartilage and adjacent to the lower border of the hyoid bone, was flipped over to cover the frontal laryngeal defect in the same way as one flips the pages of a book. The hyoid bone was placed across the remaining laryngeal cartilages. The hyothyroid membrane should be kept relatively tight because a loose membrane may drop into the laryngeal cavity under negative pressure during ventilation, and this incident may lead to laryngostenosis. The remaining defects were then partially covered above the membrane with the infrahyoid muscle. Quick-setting fibrin glue (Tissucol, Immuno, Austria) was used as a sealant. A drain (non-suction type) was placed subcutaneously.

In type A cases (**Figure 2**), supraglottic defects were repaired using the thyrohyoid membrane. Subglottic defects were repaired using the infrahyoid muscle. Glottic defects were closed using the hyoid bone. In type B cases, glottic defects were mainly repaired with the thyrohyoid membrane, and the subglottic defects were covered with the infrahyoid muscle and the hyoid bone (**Figure 3**).

#### Preoperative and postoperative assessment

The following outcome measures were prospectively recorded: morbidity and mortality (complications), oncological safety, and functional outcome.

## Results

### Functional outcome

No patient had postoperative dyspnea or dysphagia. No patient died during the postoperative course. The four patients received voice training very early after the procedure. Their vocal qualities were raspy and slightly deeper compared with their preoperative condition. The volume was weak when they made a call. The short-term functional recovery of the patients was normal (**Table 1**).

### Potential surgery-related complications

No postoperative failure of healing caused by wound infection, seroma, major subcutaneous emphysema, laryngocele, hematoma, cervical skin necrosis, laryngeal chondritis with fistula formation, laryngeal stenosis, or secondary stenosis was observed in any of the four patients.

### Oncologic results

The follow-up periods for the four patients were 43, 41, 38, and 28 months, respectively. Case one, case two and case four did not suffer from tumor recurrence during the follow-up period. Case three underwent total laryngectomy because of tumor recurrence in the paraglottic space, but no evidence of further tumor recurrence was found at the final assessment.

## Discussion

With developments in chemoradiotherapy, laser resection, and transoral robotic surgery, partial laryngectomy has not been considered as the preferred method but remains a good alternative technique of organ preservation protocols. The procedure usually provides good local control of diseases and a satisfactory functional outcome^15^^-^^17^. However, partial laryngectomy is mainly recommended for selected patients with T_3_ or T_4a_ tumor in glottic or subglottic area.

Tucker *et al.*^18^ used the epiglottis in laryngeal cavity reconstruction because the epiglottis provides support for the laryngeal cavity and inhibits excessive local granulation, thereby preventing laryngostenosis. However, this type of reconstruction alters the normal position of the epiglottis and impairs swallowing. By contrast, the use of a combined muscle-pedicle hyoid bone flap in laryngeal reconstruction provides support for the laryngeal cavity and prevents altering the position of the epiglottis. As a result, epiglottic function and swallowing are preserved. The major feature of our technique involves the use of the thyrohyoid membrane to repair laryngeal mucosa defect^19^. As a type of fascia, the thyrohyoid membrane has numerous advantages compared with other grafts^20^^,^^21^. This membrane is sufficiently large to cover defects of various sizes, easy to prepare, and adaptable to recipient sites. The thyrohyoid membrane is also resistant to saliva, infection, motion, and irradiation; this procedure also provides a high survival rate because the membrane is poorly vascularized and has low metabolism^22^^-^^24^. The combined muscle-pedicle hyoid bone and thyrohyoid membrane flap, as a chimeric flap, prevents excessive local granulation, thereby reducing the incidence of laryngotracheal stenosis, compared with muscle-pedicle hyoid bone flaps. This combination is a reliable graft to perform one-stage repair of laryngotracheal defects, providing effective repair of the mucosa and cartilage support.

In 2004, Cansiz *et al.*^14^ reported the use of muscle-pedicle hyoid bone flaps (without the thyrohyoid membrane) to reconstruct laryngeal or tracheal defects. Furthermore, four of five cases with T_3_ laryngeal carcinoma and 14 of 17 cases with laryngotracheal stenosis were decannulated at an average of 21.4 days after reconstruction. After decannulation, their respiration, speech quality, and swallowing were adequate. However, three patients (3/23, 13.6%) suffered from laryngotracheal stenosis after reconstruction. In our study, the short-term functional recovery of the four patients was normal. No patient died during the postoperative course. No postoperative dyspnea, dysphagia, and failure of healing caused by wound infection, cervical skin necrosis, or laryngeal stenosis were observed in any of the patients. Three patients did not suffer from tumor recurrence during the follow-up period. The mean duration of tracheostomy tube (3.5 d) and the nasogastric tube (7 d) in our study were shorter than those in the report of Cansiz *et al.*^14^.

The current study is a case series study describing a technique performed on only four patients; however, procedure-related complications and functional outcomes were expected. This outcome may be attributed to several advantages: (1) The hyoid bone has a high survival rate^25^ because a stable blood supply is well preserved in the flap; (2) The hyoid bone is sufficiently long to have a large cross-section that supports an extended laryngeal cavity for ventilation^26^^,^^27^; (3) The epiglottis is retained in place without “pull down” and its function is preserved; the same results are observed in the arytenoids and the cricoarytenoid joints. The cricoarytenoid unit relies on the action of the cricoarytenoid joint, which allows the arytenoid cartilage to function; (4) The use of a thyrohyoid membrane flap to reconstruct the frontal lateral laryngeal cavity prevents excessive local granulation. The thyrohyoid membrane flap is also sufficiently long to repair defects of the thyroid cartilage and the cricothyroid membrane; (5) Reconstruction does not affect the outcome of postoperative radiotherapy. In our patient series, two patients who received postoperative radiotherapy had satisfactory survival and few complications.

However, the technique also has limitations. The flap is suitable for patients who have undergone extensive vertical partial laryngectomy or frontal partial laryngectomy. At least the posterior one-third of the bilateral thyroid ala or the posterior half of the cricoid cartilage should be preserved to perform hyoid bone fixation and create a bony support for the laryngeal cavity. The pre-epiglottic space should be free of tumor to repair the mucosal defect of the thyrohyoid membrane. Laryngotracheal defect should also be limited above the level of the first tracheal ring, which is limited by the length of the thyrohyoid.

## Conclusion

The combined muscle-pedicle hyoid bone and thyrohyoid membrane flap is a reliable graft that can be used in one-stage repair of laryngotracheal defects, providing a cartilage support and an effective repair of the mucosa. Vocal quality, swallowing function, and ventilation after the procedure were favorable. However, the number of cases in this study was very small to perform statistical analysis. A large sample should be enrolled in future studies to further evaluate the importance of this flap.
